# The Landscape of Cutaneous T-Cell Lymphoma (CTCL) in the Middle East and North Africa (MENA) and the Establishment of the MENA CTCL Working Group

**DOI:** 10.3390/cancers16193380

**Published:** 2024-10-02

**Authors:** Rasha Abdel Tawab, Jihan Rajy, Salah Abdallat, Muna Abdula Almurrawi, Khalil Al Farsi, Jehad Alassaf, Hussni Alhateeti, Atlal Al Lafi, Reem El Bahtimi, Abdulhadi Jfri, Chalid Assaf

**Affiliations:** 1 Kuwait Cancer Control Centre, Kuwait City 13001, Kuwait; rabdeltawab@moh.gov.kw; 2 As’ad Al Hamad Dermatology Center, Ministry of Health, Kuwait City 13001, Kuwait; jihanrajy@yahoo.com (J.R.); lalta_k@hotmail.com (A.A.L.); 3 Jordan Royal Medical Services, King Hussein Medical Center, Amman 11855, Jordan; salah_ab@jrms.gov.jo (S.A.); jehad.alassaf@jrms.gov.jo (J.A.); 4 Dr. Muna Almurrawi Medical Center, Abu Dhabi, United Arab Emirates; dralmurrawi2012@gmail.com; 5 Sultan Qaboos University Hospital, Muscat 123, Oman; khalilf@squ.edu.om; 6 Division of Haematology and Oncology, Sheikh Shakhbout Medical City (SSMC), Abu Dhabi, United Arab Emirates; dr.h.alhateeti@gmail.com; 7 International Dermpath Consult, FZ LLC, Dubai, United Arab Emirates; reemelbahtimi@gmail.com; 8 College of Medicine, King Saud Bin Abdulaziz University for Health Sciences, Jeddah 21577, Saudi Arabia; jfria@ksau-hs.edu.sa; 9 King Abdullah International Medical Research Center, Jeddah 21589, Saudi Arabia; 10 Division of Dermatology, Department of Medicine, Ministry of the National Guard-Health Affairs, Jeddah 21577, Saudi Arabia; 11 Helios Klinikum Krefeld, 47805 Krefeld, Germany; 12 Institute of Molecular Medicine, Medical School Hamburg, 20457 Hamburg, Germany

**Keywords:** MENA, CTCL, mycosis fungoides, epidemiology, hypopigmented MF, hyperpigmented MF, pediatric MF, MENA working group

## Abstract

**Simple Summary:**

Cutaneous T-cell lymphomas (CTCLs) represent a heterogeneous group of rare extranodal non-Hodgkin lymphomas with variable clinical presentation. In the Middle East and North Africa (MENA region), where darker skin colors are more common than in the West, CTCL generally presents at a younger age and with distinct clinical features that necessitate special expertise and management across disciplines: rare forms of CTCL are more common (hypo- and hyperpigmented mycosis fungoides (MF)) and a higher prevalence of pediatric MF is noticed.

**Abstract:**

The high cancer burden in the Middle East and North Africa (MENA region) is coupled with an increasing cancer incidence. While the MENA region constitutes 6% of the world’s population, it remains underrepresented in clinical trials. Cutaneous T-cell lymphomas (CTCLs) represent a heterogeneous group of rare extranodal non-Hodgkin lymphomas with variable clinical presentation. In the MENA region, where darker skin colors are more common than in the West, CTCL generally presents at a younger age and with distinct clinical features that necessitate special expertise and management across disciplines: rare forms of CTCL are more common (hypo- and hyperpigmented MF) and a higher prevalence of pediatric MF is noticed. The multidisciplinary approach to cancer management is growing worldwide and is necessary for the comprehensive management of CTCL. The MENA CTCL group was established with the aim of creating a collaborative environment for the diagnosis and treatment of CTCL in the region. Its first meeting was held in May 2023. The group plans to increase the global representation of the MENA region and establish CTCL registries and patient advocacy groups in the region.

## 1. Introduction

**Figure 1:**
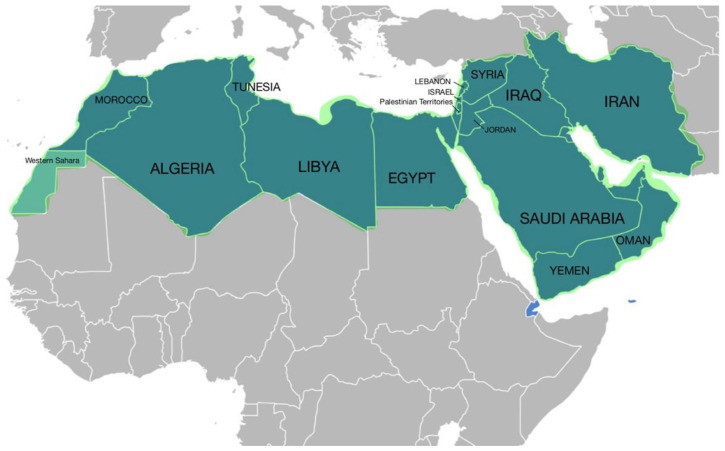
The Middle East and North Africa (MENA) is a geographic region, which comprises the Middle East and North Africa together.

The Middle East and North Africa (MENA) is the geographic region extending between Iran in the east and Morocco in the west, including 20 countries. The MENA population is estimated to be around 500 million people, constituting around 6% of the world’s population (Figure 1) [1].

While the cancer burden is a global concern, it remains much higher in low- and middle-income countries than in other parts of the world [2]. Nevertheless, by 2030 [3], there is projected to be a more than 60% increase in the cancer burden in these countries. A study by Hofmarcher et al. included data from eight MENA countries between the years 2000 and 2019 and found the increase in the cancer incidence to be 8% in Lebanon, 14% in Jordan, 65% in the UAE, and 104% in Algeria. This increase was mainly attributed to three factors: the aging population (demographic changes), lifestyle changes, and increased rates of cancer screening [4].

Despite having a wide pool of patients, the MENA region still falls behind in terms of participation in global clinical trials—including oncology trials. While the MENA region includes around 6% of the global population, it only participates in around 3% of clinical trials worldwide [5].

Cutaneous T-cell lymphomas (CTCLs) represent a heterogeneous group of rare extranodal non-Hodgkin lymphomas, with the most common subtypes being mycosis fungoides (MF) and Sézary syndrome. CTCL is characterized by the localization of neoplastic T-lymphocytes to the skin without evidence of extracutaneous disease at the time of diagnosis [6]. While the pathogenesis of CTCL is not fully understood, some studies suggest a potential role of environmental exposure [7]. The CTCL incidence in the USA is estimated to be 0.64 to 0.87 per 100,000 person-years [7].

The clinical presentation of CTCL is highly variable, often including visible skin changes such as patches, plaques, tumors and/or erythroderma [8]. In addition to the medical history and physical exam, the initial workup for patients with suspected CTCL includes multiple tests that assist in the differential diagnosis of CTCL, identifying its subtype, as well as disease staging, including biopsy of the affected skin, molecular analysis to detect clonal T-cell receptor (TCR) gene rearrangements, assessment of peripheral blood involvement, among others [9]. The International Society for Cutaneous Lymphomas (ISCL) and EORTC developed the TNMB classification and clinical staging of CTCL, which considers T (skin), N (node), M (visceral), and B (blood involvement) [9]. Early-stage MF is limited to the skin; however, it may progress to skin nodules and/or involve extracutaneous sites such as the lymph nodes, blood or visceral organs [10]. As the disease advances, survival is greatly reduced. Agar et al. reported a median overall survival of 4.7–1.4 years for MF patients with stage IIB–IVB disease [11].

The updated 2023 EORTC consensus for the treatment of mycosis fungoides/Sézary syndrome includes recommendations for the first- and second-line treatment by disease stage, while acknowledging that the choice of treatment would depend on the clinical presentation and treatment availability in each individual case [12]. Table 1 summarizes the recommendations for MF treatment by line of therapy and disease stage.

Although the treatment options have increased in recent years, e.g., with the approval of chlormethine gel or, for the advanced stage, the highly effective antibodies mogamulizumab and brentuximab vedotin, the availability of several drugs is limited in many countries in the MENA region. There is a huge difference regarding the availability of first-line treatments, e.g., interferon and retinoids, as well as second-line treatments, e.g., the antibody-based treatments, from country to country. As an example, mogamulizumab or brentuximab vedotin are currently unavailable or not covered by medical insurance in Egypt. On the other hand, all the drugs listed in the guidelines, including the antibody-based treatments, are available in Kuwait and Saudi Arabia, indicating the difference in resources in the countries in the MENA region [13].

The purpose of this paper is to clarify the local difficulties in the diagnosis and treatment of cutaneous T-cell lymphomas (CTCLs) in the Middle East and North Africa (MENA) area. It also aims to highlight the objectives and projects of the MENA CTCL working group in tackling these issues and encouraging teamwork for better patient care.

## 2. CTCL in the MENA Region

**Figure 2:**
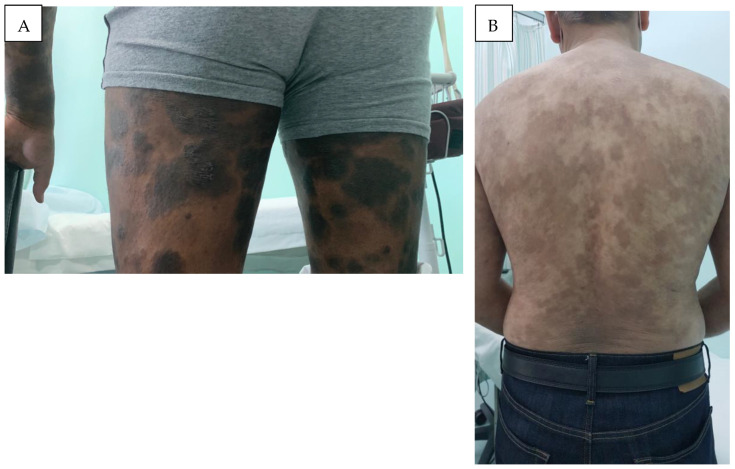
Hyperpigmented MF: (**A**) widespread hyperpigmented infiltrated scaly plaques (MF IB), and (**B**) generalized slightly scaly hyperpigmented patches of mycosis fungoides on the trunk (MF IB).

**Figure 3:**
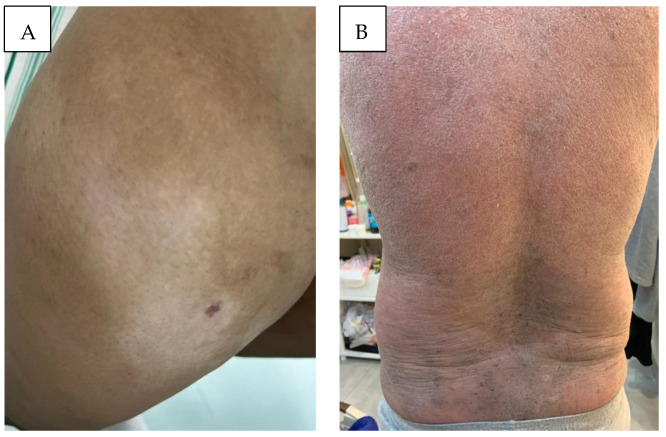
(**A**) Hypopigmented MF: localized hypopigmented patches of mycosis fungoides, restricted to the sun-protected area of the thigh. (**B**) Erythrodermic mycosis fungoides: scaly infiltrated melanoerythroderma covering >90% of the body surface area (MF III).

CTCL in the MENA region exhibits distinct characteristics compared to Western populations in the EU and USA. The MENA region is characterized by a young average population age, and the diverse Middle Eastern populations generally exhibit darker skin tones compared to Europeans or Americans. Accordingly, there are distinct clinical features to consider in MF presenting in Middle Eastern patients. In Middle Eastern MF patients, it is noted that hyper- and hypopigmented MF (Figure 2A,B, Figure 3A) is more common, patients present at a younger age, and pediatric MF is more common, as compared to Western populations.

Erythema, for instance, appears differently on darker skin tones (melanoerythroderma, Figure 3B), which is one possible explanation why MF is diagnosed at more advanced stages and has worse outcomes in skin of color [14,15]. In addition, rare forms of MF are more common in skin of color [16].

**Figure 4:**
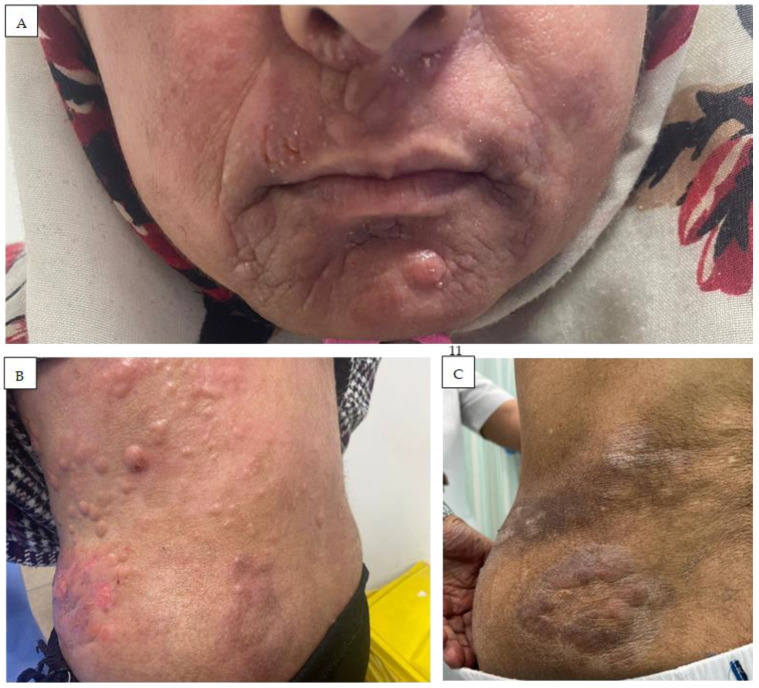
Tumor stage MF (IIB): multiple nodules and plaques, partly with erosions on the face (**A**) and trunk (**B,C**), histologically with large cell transformation.

In the MENA region, MF management is shared across different disciplines, where it tends to be managed by dermatologists in the early stages, while hemato-oncologists generally manage the stages that are more advanced, e.g., tumor stage MF (Figure 4A,B). These differences in the CTCL presentation and management highlight the need for special expertise when dealing with MF in Middle Eastern patients.

### 2.1. Studies by Country

Studies by country show differences in the clinical presentation and age of the patients (for an overview, see Table 2).

#### 2.1.1. Iran

Naeini et al. described the epidemiological characteristics of 95 primary CTCL patients diagnosed between 2003 and 2013 in Isfahan, Iran. The study highlighted the absence of a male predominance and lower age at diagnosis in this group, with a male-to-female ratio of 1:1.2 and a mean age at diagnosis of 41.78 years [17].

Another study during the same period provided clinical information on patients with MF specifically and reported the same male-to-female ratio and mean age at diagnosis. It included 86 patients, 6% of whom were children, and highlighted that 21% of cases had unusual variants of MF, including hypopigmented and poikilodermatous MF [18].

Nasimi et al. studied 30 cases of pediatric MF and concluded that persistent hypopigmented, purpuric and papulosquamous lesions in children is suggestive of MF as a differential diagnosis. The study reported excellent results with phototherapy in this patient group [19].

#### 2.1.2. UAE

Hamodat et al. conducted a retrospective review and identified 40 patients with mycosis fungoides between 2013 and 2019. The male-to-female ratio was 1.22 and the median age at diagnosis was 47 years. In addition, 20% of patients presented with hypopigmented patches and 12.5% with hyperpigmented patches [20].

#### 2.1.3. KSA

One study included 34 cases of MF diagnosed between 2011 and 2016 and reported that the hypopigmented type was the most common type of MF in the study, affecting a younger age group. The study reported a good response to phototherapy NB-UVB combined with topical corticosteroids in the study group [21].

Another study from Riyadh included 125 CTCL patients (2010–2016) and reported that the hypopigmented type occurred in 15.2% of cases. The male-to-female ratio was 1.36, the median age at diagnosis was 41 years, and 13% of patients were younger than 20 years of age [22].

Alghubaywi et al. enrolled 73 patients diagnosed with MF between 2016 and 2022 and reported an early onset of disease, with a mean age of 44 years, a slightly higher prevalence in females, with a male-to-female ratio of 1.3:1, and a higher prevalence of hypopigmented MF (20.5%) [23].

Another study included 66 skin biopsies generated from 58 patients with suspected/early patch stage MF between 2002 and 2006 and concluded that observing the hyperconvoluted dermal and epidermal lymphocytes, among other parameters, plays an important role in the histopathological diagnosis of early MF lesions and their discrimination from inflammatory simulators [24].

#### 2.1.4. Morocco

One study conducted on 114 MF cases diagnosed from 1993 to 2022 reported a male-to-female ratio of 2.56, a median age at diagnosis of 56 years, and a high proportion of classic MF with a favorable prognosis [25].

#### 2.1.5. Kuwait

One study included 193 MF cases diagnosed between 1991 and 2006 and reported a male-to-female ratio of 2:1, a mean age at diagnosis of 35 years, and a proportion of patients diagnosed by the age 20 years of 16%. In addition, 22% of patients had a pure hypopigmented variant, and they were observed to have a younger mean age at diagnosis (27.6 years) compared to other MF cases (38.1 years). The calculated annual incidence rate of MF in Kuwait was 0.43 cases per 100,000 persons [26].

Another study included 738 subjects registered with non-Hodgkin’s lymphoma between 1998 and 2006 in the population-based cancer registry at the Kuwait Cancer Control Center. The study reported that the prevalence of MF was 9.3% in this group [27].

**Figure 5:**
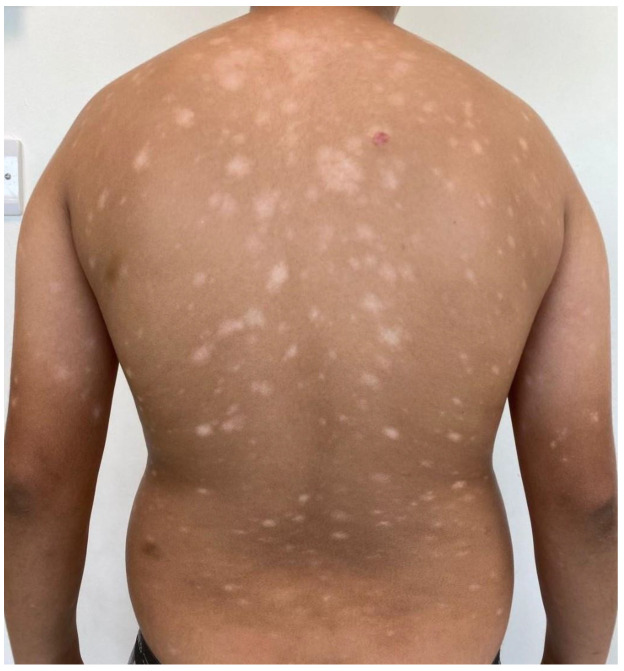
Pediatric MF, hypopigmented variant: generalized hypopigmented macules and patches in a 13-year-old male (MF IB).

Nanda et al. studied the clinico-epidemiologic features of juvenile onset MF in Kuwait and included 36 patients diagnosed between 1991 and 2009. Juvenile onset MF constituted 16.6% of the total number of MF patients and the age-adjusted incidence rate of MF in children and adolescents among the total population was 0.29/100,000 persons/year. The study reported a male-to-female ratio of 1.25:1 and a mean age at onset of disease of 9 years and at diagnosis of 13 years. Patients had a predominantly hypopigmented presentation (56% of the cases, Figure 5) [28].

#### 2.1.6. Egypt

Abdel-Halim et al. studied the frequency of hypopigmented MF in a cohort of 100 Egyptian patients presenting with hypopigmented lesions involving the trunk (with or without other sites’ involvement). The frequency of hypopigmented MF was found to be 16%. Hypopigmented MF was significantly associated with a progressive disease course, affection of the distal upper limbs, proximal lower limbs, large-sized lesions (>5 cm), a well-defined margin, scaliness, erythema, atrophy, and mottled pigmentation, as compared to other hypopigmented disorders [29].

#### 2.1.7. Jordan

A retrospective study analyzing the clinical and pathological features of MF included 63 patients diagnosed between 2000 and 2015 and reported a male-to-female ratio of 2.15:1, a mean age at diagnosis of 45 years, and the following clinical variants: 39.6% classical MF, 11% hypopigmented MF, 9.5% poikilodermatous MF, and 3.1% hyperpigmented MF [30].

### 2.2. Multidisciplinary Approach in CTCL Management

The multidisciplinary team approach to disease management is growing in popularity across different therapeutic areas, especially oncology, and CTCL is no exception [30]. Vitiello et al. suggest that a multidisciplinary team, including dermatologists, hematologists, oncologists, and support staff, should be involved in the management of CTCL [31]. The authors suggest that while dermatologists usually handle treatment in the early phases, they should still be involved in CTCL management even in advanced stages where systemic therapies are needed. Dermatologists’ involvement in CTCL management is necessary considering their knowledge of the topical and systemic therapies for CTCL and the use of the TNMB classification and staging system, their ability to perform frequent skin examinations to monitor the response to treatments and toxicities, and their role in the evaluation and treatment of pruritus, skin infections, and wound care [8]. A review by Dai et al. suggested that the multidisciplinary approach to diagnosing and treating MF and SS should integrate anticancer therapies with skincare and bacterial decolonization to ensure comprehensive management, which should typically be performed by a multidisciplinary team that includes dermatologists, oncologists, radiation oncologists, and bone marrow transplant specialists [32].

### 2.3. Establishment of the MENA CTCL Group

The inaugural meeting of the MENA CTCL group took place in Dubai on 6 May 2023. This meeting marked the beginning of a collaborative interdisciplinary effort to address the challenges faced in the diagnosis and treatment of CTCL in the MENA region. The CTCL MENA group’s aims and tasks are as follows. (1) To establish regular communication between CTCL experts in the MENA region in the form of regular interdisciplinary tumor boards. The board meetings will provide a platform for participants to present and discuss challenging CTCL cases. Collaborative discussions among board members will contribute to the accuracy and effectiveness of diagnoses and treatment plans, ultimately improving the quality of care for CTCL patients in the MENA region. (2) To involve different multidisciplinary actors (dermatologists, hematologists, oncologists, pathologists, as well as radiotherapists) in the care of patients. (3) To increase representation of the MENA region, e.g., in guidelines, publications, clinical studies, etc., considering the special clinical ethnic features and therapeutic limitations in the management of CTCL patients. The board plans to document and publish regional-specific data, such as patient characteristics (age, skin color), clinical cases, and guidelines. These publications will contribute to the body of knowledge regarding CTCL and improve future treatment approaches in this region, including establishing CTCL registries and establishing CTCL patient advocacy groups in the MENA region.

## 3. Conclusions

In conclusion, the heterogenous nature of CTCL, in addition to the unique ethnic features of the population in the MENA region, such as hypo- and hyperpigmented MF and increased pediatric cases, contributes to the distinct clinical features of CTCL specific to this population. These differences from Western patients highlight the need for special expertise and multidisciplinary management of CTCL. The MENA CTCL group was established with the objective of improving the diagnosis and treatment of CTCL in the region through creating a collaborative environment and increasing the global representation of the MENA region. Further research is needed to elucidate epidemiological trends, establish CTCL registries, and develop tailored diagnostic and therapeutic strategies.

## Figures and Tables

**Table 1:** Treatment recommendation for MF, modified according to [12].

Stage	First Line	Second Line
IA, IB, and IIA	Expectant policy (mainly T1a) Skin-directed therapy: Topical corticosteroids (mainly T1a and T2a) Topical chlormethine nbUVB (mainly T1a and T2a) PUVA Localized RT (for localized MF, including pagetoid reticulosis)	Systemic therapies Retinoids PEG-IFN-α TSEB (mainly T2b) Brentuximab vedotin Mogamulizumab Low-dose MTX
IIB	Systemic therapies Retinoids PEG-IFN-α TSEB Brentuximab vedotin Mogamulizumab Monochemotherapy (pegylated liposomal doxorubicin, gemcitabine) Low-dose MTX Localized RT	(Poly-)chemotherapy Brentuximab vedotin Mogamulizumab Allogeneic stem cell transplantation
IIIA and B	Systemic therapies Retinoids PEG-IFN-α ECP Brentuximab vedotin Mogamulizumab Low-dose Methotrexate (MTX) TSEB	Monochemotherapy (gemcitabine, pegylated liposomal doxorubicin) Brentuximab vedotin Mogamulizumab Allogeneic stem cell transplantation
IVA and IVB	Chemotherapy (gemcitabine, pegylated liposomal doxorubicin,) Radiotherapy (TSEB and localized) Brentuximab vedotin Mogamulizumab Alemtuzumab (mainly in B2) CHOP and CHOP-like polychemotherapy Allogeneic stem cell transplantation	

MF: mycosis fungoides; NBUVB: narrowband ultraviolet B; PUVA: psoralen and ultraviolet A; TSEB: total skin electron beam; PEG-IFN: interferon (currently only the pegylated form of interferon-alpha is available); SDT: skin-directed therapy; MTX: methotrexate; ECP: extracorporeal photopheresis, RT: radiotherapy.

**Table 2:** Epidemiological and clinical insights into CTCL: a multinational retrospective analysis.

Study (1st Author et al.)	Study Type	Country	Patients Number	Study Endpoints	Results
Naeini et al. (2014) [17]	Epidemiological descriptive study	Iran	95 primary CTCL patients diagnosed between 2003 and 2013 in Isfahan	Epidemiological characteristics of primary CTCL patients in Isfahan, Iran	Absence of male predominance, lower age at diagnosis (male-to-female ratio of 1:1.2, mean age at diagnosis of 41.78 years)
Naeini et al. (2015) [18]	Clinical descriptive study	Iran	86 patients with MF diagnosed between 2003 and 2013 in Isfahan	Clinical information on patients with MF, including unusual variants	Male-to-female ratio of 1:1.2, mean age at diagnosis similar to primary CTCL patients (41.78 years), 6% children, 21% unusual variants (hypopigmented and poikilodermatous MF)
Nasimi et al. (2020) [19]	Clinical study	Iran	30 cases of pediatric MF	Evaluation of hypopigmented, purpuric, and papulosquamous lesions in pediatric MF	Suggestive features for MF diagnosis in children, excellent results with phototherapy
Hamodat et al. (2021) [20]	Retrospective review	UAE	40 patients with mycosis fungoides between 2013 and 2019	Epidemiological characteristics of mycosis fungoides patients in the UAE	Male-to-female ratio of 1.22, median age at diagnosis of 47 years, 20% with hypopigmented patches, 12.5% with hyperpigmented patches
Alojail et al. (2022) [21]	Retrospective review	KSA	34 cases of MF diagnosed between 2011 and 2016	Characteristics and response to phototherapy of MF patients	Hypopigmented MF most common, good response to NB-UVB combined with topical corticosteroids
Binamer (2017) [22]	Retrospective review	KSA	125 CTCL patients diagnosed between 2010 and 2016	Epidemiological characteristics of CTCL patients	Male-to-female ratio of 1.36, median age at diagnosis of 41 years, 15.2% hypopigmented type
Alghubaywi et al. (2023) [23]	Retrospective review	KSA	73 patients diagnosed with MF between 2016 and 2022	Epidemiological characteristics of MF patients	Mean age of onset of 44 years, female-to-male ratio of 1.3:1, 20.5% hypopigmented MF
Arafah et al. (2012) [24]	Histopathological study	KSA	58 patients with suspected/early patch stage MF between 2002 and 2006	Histopathological diagnosis of early MF lesions	Importance of hyperconvoluted dermal and epidermal lymphocytes in diagnosis
Titou et al. (2023) [25]	Retrospective study	Morocco	114 MF cases diagnosed from 1993 to 2022	Epidemiological characteristics of MF patients	Male-to-female ratio of 2.56, median age at diagnosis of 56 years, high proportion of classic MF
Alsaleh et al. (2010) [26]	Retrospective review	Kuwait	193 MF cases diagnosed between 1991 and 2006	Epidemiological characteristics of MF patients	Male-to-female ratio of 2:1, mean age at diagnosis of 35 years, 16% diagnosed before age 20, 22% pure hypopigmented variant
Ameen et al. (2010) [27]	Population-based study	Kuwait	738 subjects with non-Hodgkin’s lymphoma registered between 1998 and 2006	Prevalence of MF among non-Hodgkin’s lymphoma patients	Prevalence of MF was 9.3% in the study population
Nanda et al. (2010) [28]	Epidemiological study	Kuwait	36 patients diagnosed with juvenile onset MF between 1991 and 2009	Clinico-epidemiologic features of juvenile onset MF	Juvenile onset MF constituted 16.6% of total MF cases, male-to-female ratio of 1.25:1, mean age at onset of 9 years, predominantly hypopigmented presentation (56% of cases)
Abdel-Halim et al. (2015) [29]	Observational study	Egypt	100 Egyptian patients presenting with hypopigmented lesions	Frequency and association of hypopigmented MF	Frequency of hypopigmented MF was 16%, significantly associated with progressive disease course and specific clinical features
Al-Tarawneh (2018) [30]	Retrospective study	Jordan	63 patients diagnosed between 2000 and 2015	Clinical and pathological features of MF	Male-to-female ratio of 2.15:1, mean age at diagnosis of 45 years, various clinical variants observed
